# Peer Review of “A Physical Activity Mobile Game for Hematopoietic Stem Cell Transplant Patients: App Design, Development, and Evaluation”

**DOI:** 10.2196/28339

**Published:** 2021-04-13

**Authors:** Abigail Fisher

**Affiliations:** 1 Physical Activity and Health Department of Behavioural Science and Health University College London London United Kingdom

**Keywords:** cancer, mobile app, gamification, bone marrow transplant, alpha testing, physical activity

*This is a peer-review report submitted for the paper "A Physical Activity Mobile Game for Hematopoietic Stem Cell Transplant Patients: App Design, Development, and Evaluation."*

## Round 1

### Comments for Authors/Editors

#### General Comments

1. This was a novel and interesting manuscript [1] on the development and user evaluation of a walking app for hematopoietic stem cell transplant (HSCT) patients. The main comment is that clarity on who comprised the usability samples (survey respondents, initial usability testers, additional usability evaluators), and who specifically the target sample for the game is (HSCT patients can be fairly diverse), would enhance the paper.
2. In general, the conducted and planned usability testing seemed heavy on the expert testing and light in terms of planned testing with patients. In addition, the focus appeared to be very much on usability testing, without much acknowledgment that there would be a need in the future to test the impact on walking behavior.
3. While there is no doubt that expert usability testing is important, and it is nice to see clear descriptions of the early development processes, it does not seem sufficient to then do a short usability test with patients and release the app to the public.
4. It would be good to acknowledge that rigorous evaluation (including feasibility, acceptability, and measured impact on walking) would be required prior to release. I am sure that this is planned and has been considered, but perhaps a flowchart or figure/table outlining each of the development steps, the samples involved in these steps, and details on a trial exploring the impact on walking within the target sample might bring clarity.

#### Specific Comments

These are mostly for clarity rather than any issue with the study.

##### Abstract

1. Minor, but rather than “the aim of this paper,” replace with “the aim of this study” or “the aim of this paper was to describe….”
2. Make it clear that the paper describes only the evaluation, rather than a behavioral evaluation (ie, impact on physical activity), and that the evaluation took place with game development experts and clinicians rather than patients. This was not clear until quite far into the methodology. Some of the results (eg, “moving tiles”) lack context in the abstract.

##### Introduction

1. In general, this section is well written but could have included more details on interventions that have tried to promote physical activity in HSCT patients in other contexts to give a full lay of the land.
2. While the hypotheses around the reasons why the app might encourage walking are logical, it would have been good to include some references to support these, and some of the justification for app content might have been better placed in the *Methods* section.
3. It might have been good to give an indication of the likely target sample(s) (eg, in terms of age) and information on smartphone ownership in these groups because the population (particularly age range) can be very diverse. It would be interesting to get a sense of whether there was a particular target demographic in mind for this game.

##### Methods

1. Tied to the comment above, it was nice to see some formative survey work. Again, it would be interesting to get an idea of the age range and other demographics of the response sample and whether these are exactly in line with the target sample for this new game (eg, Candy Crush tends to be more popular among specific demographics).
2. Per the comment above, it was not immediately clear that this paper would describe testing with game experts and a nurse, rather than patients. This could be outlined early in the methodology.
3. Table 1 looks like it discloses emails; this should be removed if they are genuine.
4. Aside from replicating the model of Candy Crush, was there any consideration for, or attempt to, include some of the key behavior change techniques that are important for physical activity change (eg, goal setting, self-monitoring, feedback)? I can imagine they are probably in there by default, but it would be nice to see which behavior change techniques map to which game features and if there was any consideration of a theoretical basis in the app development process.
5. The qualitative analysis is not mentioned until the *Data Analysis* section—what was the purpose and how was it carried out? It is worth acknowledging in the *Methods* to provide context. In addition, I found the sentence describing the qualitative analysis difficult to follow. Could this be simplified?
6. The sentence on step counters, “To improve the accuracy of our step counters of our designed WW, we recruited 5 additional usability evaluators who were nursing informatics graduate students,” could have been described in the *Methods* section, as it came out of the blue in the *Results*. In general, clarity on who comprised the usability samples (survey respondents, testers, additional usability evaluators, and the actual target sample for the game) would enhance the paper.

##### Discussion

1. Discussion and conclusion are very short—it would have been good to describe more current findings in relation to other relevant studies. Another sample of 30 individuals (students and programmers) was described here, which seems like it might be planned work, but this was not entirely clear. Perhaps a flowchart or table with all of the planned steps and samples involved would be useful.
2. Per the general comment at the start, the focus seems very expert heavy, with only a brief evaluation with patients and a strong focus on usability rather than the impact on walking behavior. It would be important to trial the app in patients to determine whether it has an impact on walking behavior. To what extent does it matter whether people find it usable and like it if it does not actually change the target behavior?

## Abbreviations

HSCT: hematopoietic stem cell transplant
